# IP-10-Mediated T Cell Homing Promotes Cerebral Inflammation over Splenic Immunity to Malaria Infection

**DOI:** 10.1371/journal.ppat.1000369

**Published:** 2009-04-03

**Authors:** Catherine Q. Nie, Nicholas J. Bernard, M. Ursula Norman, Fiona H. Amante, Rachel J. Lundie, Brendan S. Crabb, William R. Heath, Christian R. Engwerda, Michael J. Hickey, Louis Schofield, Diana S. Hansen

**Affiliations:** 1 The Walter and Eliza Hall Institute of Medical Research, Parkville, Victoria, Australia; 2 Department of Medical Biology, The University of Melbourne, Parkville, Victoria, Australia; 3 Centre for Inflammatory Diseases, Monash University, Department of Medicine, Monash Medical Centre, Clayton, Victoria, Australia; 4 Queensland Institute of Medical Research, Herston, Queensland, Australia; 5 Burnet Institute, Melbourne, Victoria, Australia; 6 Department of Microbiology and Immunology, The University of Melbourne, Parkville, Victoria, Australia; London School of Hygiene and Tropical Medicine, United Kingdom

## Abstract

*Plasmodium falciparum* malaria causes 660 million clinical cases with over 2 million deaths each year. Acquired host immunity limits the clinical impact of malaria infection and provides protection against parasite replication. Experimental evidence indicates that cell-mediated immune responses also result in detrimental inflammation and contribute to severe disease induction. In both humans and mice, the spleen is a crucial organ involved in blood stage malaria clearance, while organ-specific disease appears to be associated with sequestration of parasitized erythrocytes in vascular beds and subsequent recruitment of inflammatory leukocytes. Using a rodent model of cerebral malaria, we have previously found that the majority of T lymphocytes in intravascular infiltrates of cerebral malaria-affected mice express the chemokine receptor CXCR3. Here we investigated the effect of IP-10 blockade in the development of experimental cerebral malaria and the induction of splenic anti-parasite immunity. We found that specific neutralization of IP-10 over the course of infection and genetic deletion of this chemokine in knockout mice reduces cerebral intravascular inflammation and is sufficient to protect *P. berghei* ANKA-infected mice from fatality. Furthermore, our results demonstrate that lack of IP-10 during infection significantly reduces peripheral parasitemia. The increased resistance to infection observed in the absence of IP-10-mediated cell trafficking was associated with retention and subsequent expansion of parasite-specific T cells in spleens of infected animals, which appears to be advantageous for the control of parasite burden. Thus, our results demonstrate that modulating homing of cellular immune responses to malaria is critical for reaching a balance between protective immunity and immunopathogenesis.

## Author Summary

About 2.5 million people die of severe *Plasmodium falciparum* malaria every year. Experimental evidence from human studies and animal models indicates that severe disease syndromes arise in many organs through the sequestration of parasitized erythrocytes in vascular beds and the resulting recruitment of inflammatory leukocytes. Thus in this infection, cell-mediated immune responses appear to play a dual role by mediating protection against the parasite and also contributing to pathogenesis. Using a rodent model of cerebral malaria, we have previously found that during infection, inflammatory leukocytes are recruited to the brain via the CXCR3 trafficking pathway. Here we investigated whether blockade of the CXCR3 ligand, IP-10, alleviates brain intravascular inflammation and has an impact on the development of parasite-specific cellular immune responses involved in the control of parasitemia. We found that mice lacking IP-10 or receiving anti-IP-10 neutralizing antibodies had reduced cerebral intravascular inflammation and were protected against fatality. Inhibition of IP-10-mediated trafficking also resulted in retention of parasite-specific T cells in the spleen, facilitating control of parasite burden. Thus, IP-10-dependent trafficking critically controls the balance between pathogenic organ-specific inflammation and spleen-mediated protective immunity to malaria.

## Introduction

Malaria is one of the most serious infectious diseases in humans, infecting 5–10% of the world's population. The most severe complication caused by *Plasmodium falciparum* infection is cerebral malaria (CM), which is responsible for about 2.5 million deaths each year [1]. This neurological syndrome is characterized by the occurrence of seizures and coma [2]. Although the precise mechanism leading to cerebral disease is not fully understood, it has been suggested that sequestration of parasitised red blood cell (pRBC) in brain blood vessels induces blood flow obstruction resulting in hypoxia, haemorrhage and pathology [3].

The analysis of brain infiltrates predisposing to CM in humans is limited as it can only be deduced from post-mortem samples. Much useful evidence contributing to the understanding of disease has been provided by experimental infection with *P. berghei* ANKA. This rodent infection has many features in common with human disease and is thus a good model for some important aspects of clinical malaria [4]. A large body of work in this and other rodent models of CM demonstrated that immune responses elicited during infection play a role in the control of parasitemia but can also result in detrimental inflammation and contribute to disease induction [5],[6]. Current views support the idea that CM is caused by the combined effect of sequestration of pRBC and a strong inflammatory response mediated by cytokines such as TNF-α [7], LT-α [8], IFN-γ [9] and effector cells such as CD4^+^ [10] and CD8^+^ T cells [11],[12], NKT cells [13] and NK cells [14]. Since it is known that these cells produce cytokines that up-regulate the expression of adhesion molecules like ICAM-1, involved in the recognition of parasitic proteins expressed on pRBC, it has been proposed that this systemic inflammatory cascade exacerbates parasite sequestration. However, emerging evidence in human malaria and animal models [11],[12],[15],[16] revealed the presence of leukocytes in brain blood vessels during infection, suggesting that intravascular infiltration of these cells might result in local inflammation and could also contribute to disease induction.

Both CD4^+^ and CD8^+^ T cells have been found in brain blood vessels of CM-affected mice [11],[12]. Brain-sequestered cytotoxic CD8^+^ T cells have been shown to mediate CM via a perforin-dependent mechanism [11]. Recent work indicates that CD8^+^ T cells specific for parasite-expressed antigens are amongst those recruited to the brain during infection and are capable of mediating lethal disease [17]. Like T cells, NK cells have been found in brain blood vessels of malaria infected mice and appear to be abundant at early stages of infection [14].

The chemokine pathways responsible for leukocyte recruitment to the brain in CM have not been completely characterized. Mice deficient in CC chemokine receptor 5 (CCR5) have been reported to be either 80% resistant to *P. berghei* ANKA-mediated CM [18] or to display a delayed onset of cerebral disease symptoms [11]. In a previous study, we found that the majority of NK cells and T cells in brains of malaria-infected mice express CXC chemokine receptor 3 (CXCR3) suggesting that trafficking through this pathway is strongly associated with lymphocyte recruitment leading to cerebral disease [14]. Moreover, splenic T cells from CM susceptible but not resistant mice were found to up-regulate expression of CXCR3 and to acquire the capacity to migrate in response to CXCR3 chemokines during malaria infection, indicating that CXCR3 expression correlates with disease severity [19]. In agreement, it has been recently found that 70–80% of CXCR3^−/−^ mice are resistant to *P. berghei*-mediated CM [20],[21].

CXCR3 recognizes 3 ligands: MIG, IP-10 and I-TAC. Although all these chemokines are induced by IFN-γ, experimental evidence suggests that they play non-redundant roles in leukocyte homing [22]. These chemokines have been shown to recruit NK cells and T_H_1 cells in several inflammatory conditions and in addition to their chemotactic activity they have been shown to participate in the induction of effector immune responses. IP-10 and MIG have been found to stimulate T cell proliferation and IFN-γ production in response to alloantigen or to exogenous antigen implying a role in T_H_1 polarization [23],[24].

The role of CXCR3 chemokines in malaria has not been extensively investigated. Although it has been shown that IP-10 and MIG are up-regulated in the brain in response to infection [19],[21], their precise role in disease induction remains elusive. It has been recently shown that MIG^−/−^ and IP-10^−/−^ mice are partially resistant to *P. berghei* ANKA infection [20]. However, whether the increased survival rates to malaria infection in the absence of these chemokines result from reduced leukocyte recruitment to the brain and/or a differential induction of immune response to infection has not been examined. Moreover, experimental evidence indicates that the same cell-mediated immune responses involved in severe disease induction are also required for the control of infection [5],[6] and whether leukocyte trafficking blockade has an impact on the development of malaria-specific immunity and the control of parasite burden remains unknown. The precise understanding of these processes and their implications are highly relevant in assessing the feasibility of anti-leukocyte trafficking therapies to reduce organ-specific inflammation and fatalities associated with CM. Since recent reports identified IP-10 as a biomarker associated with mortality in *P. falciparum*-mediated CM [25],[26], in this study we thoroughly investigated the role of this chemokine during experimental CM and in the induction of parasite-specific immunity. We found that specific neutralization of IP-10 during infection reduces intravascular inflammation in brains of *P. berghei* ANKA-infected mice and is sufficient to protect from fatality. Furthermore, our data reveal that inhibition of IP-10-mediated leukocyte trafficking also results in retention of parasite-specific T cells in the spleen, which favors induction of protective immunity and facilitates control of parasite burden.

## Results

### Novel anti-IP-10 neutralizing monoclonal antibodies protect susceptible mice from *P. berghei* ANKA-mediated CM

**Figure 1:**
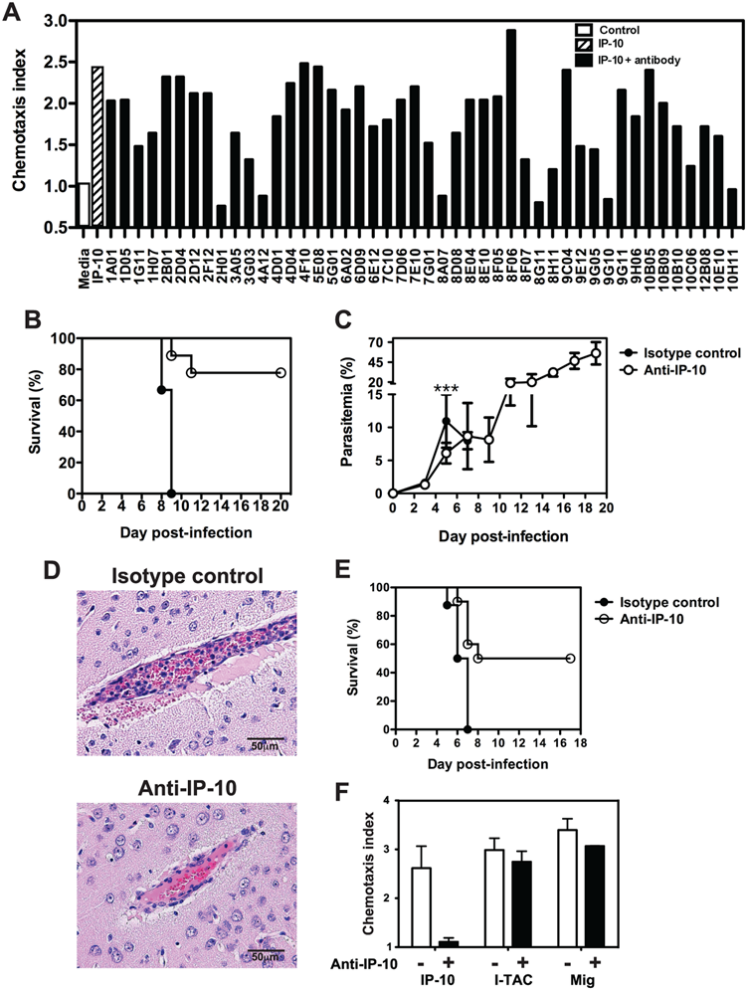
Anti-IP-10 neutralization protects susceptible mice from *P. berghei* ANKA-mediated CM. (A) Anti-IP-10 hybridoma supernatants were incubated with IP-10. Transwell inserts containing splenic T cells from malaria-infected mice were then added to the wells and chemotaxis was assessed. Bars represent chemotaxis indices. C57BL/6 mice were infected with *P. berghei*-ANKA and treated with anti-IP-10 or isotype control on days 3–9 p.i. (B) or on days 5–9 p.i. (E). Survival was monitored daily. (C) Parasitemia was determined by Giemsa-stained blood smears. Each point represents mean parasitemia±SD, ***p<0.0005 (Mann-Whitney test). Data is representative of 3 infections. (D) Histological examination of brains from *P. berghei* ANKA-infected anti-IP-10 and isotype control-treated mice. (F) Anti-IP-10 monoclonal antibody 8A7 was incubated with IP-10, MIG or I-TAC. Transwell inserts containing splenic T cells from malaria-infected mice were added to wells and chemotaxis was determined. Bars represent means of 3 samples±SEM.

To investigate the role of IP-10-mediated chemotaxis on the development of CM, we generated and screened rat monoclonal antibodies for their ability to neutralize IP-10-mediated migration of splenic T cells isolated from malaria-infected mice in *in vitro* chemotaxis assays. Six clones completely blocked IP-10-mediated chemotaxis (Figure 1A) and were selected for *in vivo* studies. In these experiments, C57BL/6 mice were challenged with *P. berghei* ANKA and on days 3–9 post-infection (p.i.) they received anti-IP-10 antibodies or an isotype control. Mice receiving anti-IP-10 antibodies were protected against CM (not shown). Treatment with clone 8A7 resulted in 80% survival (p = 0.0002) in infected animals (Figure 1B). This clone was selected for further studies. Parasitemia of 8A7-treated mice was significantly lower than in control animals during the first week of infection (Figure 1C). Although this difference was small, it was reproducible and was also evident in mice treated with other anti-IP-10 clones (not shown). Parasitemia levels rose in anti-IP-10-injected mice after treatment cessation during the second week of infection, resembling parasitic burden observed in CM-resistant mouse strains such as BALB/c [13]. No signs of CM were observed in anti-IP-10-treated mice at these late stages on infection. The diagnoses of CM were confirmed by histological examination of brains at day 6 p.i. Control mice displayed typical pathology, evidenced as high levels of vascular occlusion with pRBC and leukocytes in over 65–70% of blood vessels examined. Although around 50% of blood vessels in anti-IP-10 treated mice had signs of intravascular inflammation, the level of occlusion was clearly reduced compared to control mice (Figure 1D). To further evaluate whether anti-IP-10 treatment had therapeutic potential, *P. berghei*-infected mice were treated with this antibody after the onset of disease signs on day 5 p.i. This late administration of anti-IP-10 significantly protected susceptible mice from CM (p = 0.0062), resulting in 50% survival of infected animals (Figure 1E). Since CXCR3 chemokines share around 30% of sequence homology with each other, 8A7 specificity was tested in chemotaxis assays. 8A7 completely blocked IP-10-mediated chemotaxis but was unable to prevent migration of splenic T cells from malaria-infected mice in response to MIG or I-TAC (Figure 1F). Thus specific neutralization of IP-10 alone during infection is sufficient to protect susceptible mice from CM.

### IP-10 neutralization alleviates intravascular infiltration and does not affect parasite sequestration

**Figure 2 ppat-1000369-g002:**
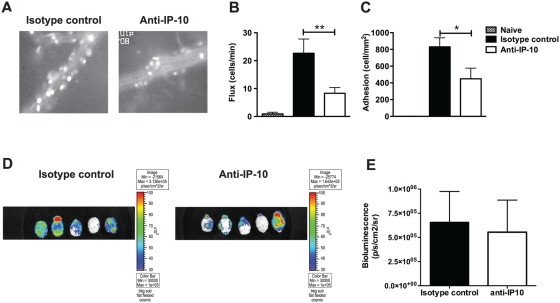
IP-10 neutralization alleviates brain intravascular infiltration. *P. berghei* ANKA-infected mice were treated with anti-IP-10 or isotype control antibodies. (A) Pial microcirculation was examined by intravital microscopy on day 5 p.i.. The number of rolling (B) and adherent (C) cells was determined. Bars represent means of 4–5 mice±SEM, *p<0.05, **p<0.01 (Mann-Whitney test). C57BL/6 mice were infected with luciferase-expressing *P. berghei* ANKA and then treated with anti-IP-10 or isotype control antibodies. (D) Brain-sequestered parasites were visualized on day 6 p.i 1 h after luciferin injection. (E) Parasite-associated bioluminescence was determined. Bars represent means of 5 samples±SD.

**Figure 3:**
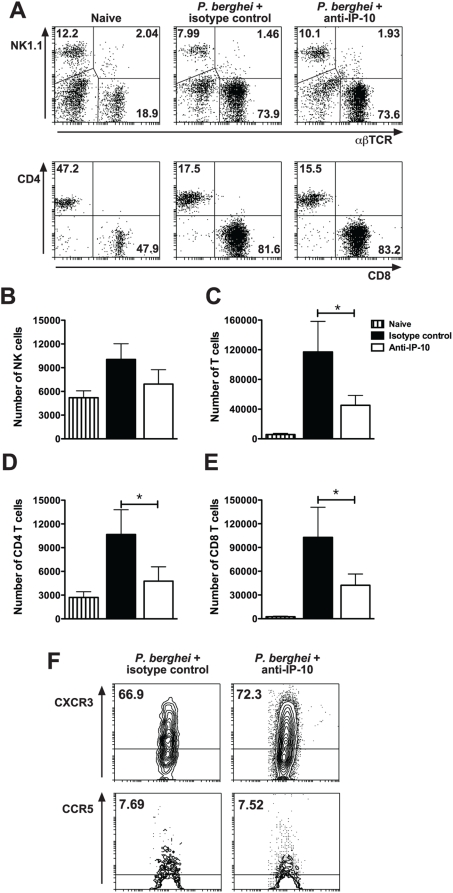
Anti-IP-10 treatment reduces T cell recruitment to the brain during malaria. C57BL/6 mice were infected with *P.berghei* ANKA and treated with anti-IP-10 or isotype control antibodies. Brain-infiltrating leukocytes were isolated on day 6 p.i., stained with fluorescent antibodies and analysed by flow cytometry. Percentage (A) and absolute number of NK cells (B), total T cells (C), CD4^+^ (D) and CD8^+^ T cells (E) were calculated. (F) CXCR3 and CCR5 expression was analysed on gated TCR^+^NK1.1^−^ cells. Each experiment is representative of 3 infections. Representative histograms and contours are shown. Bars represent means of 3 samples±SD, *p<0.05 (Mann-Whitney test).

To more accurately assess organ-specific inflammation after anti-IP-10 treatment, C57BL/6 mice were challenged with *P. berghei* ANKA, treated with 8A7 or isotype control antibody and their brain blood vessels were examined on day 5 p.i. by intravital microscopy. To that end, mice were anaesthetized, injected with rhodamine 6G to label circulating cells and pial microcirculation was observed. In contrast to naïve mice (Video S1), in which leukocyte rolling and adhesion were rarely observed, large numbers of rolling and adherent cells were observed in blood vessels of malaria-infected mice (Figure 2A and Video S2). Anti-IP-10 treatment reduced the number of rolling and adherent cells by 60% compared to isotype control-injected animals (Figure 2B,C and Video S3), indicating that this treatment alleviates intravascular infiltration during infection. Since rhodamine 6G not only labels circulating leukocytes but also pRBC [27], we sought to assess the possibility that the reduced levels of occlusion in anti-IP-10 treated mice reflected a reduction in parasite sequestration. To that end, C57BL/6 mice were infected with a transgenic *P. berghei* ANKA line that constitutively expresses luciferase [28]. Following luciferin injection on day 6 p.i., mice were sacrificed and bioluminescent parasites were observed in the brain. No significant differences were found in bioluminescence levels emerging from parasites in brains of anti-IP-10 and isotype control-treated mice (Figure 2D,E). Together these results suggest that IP-10 neutralization does not affect parasite sequestration but instead alleviates vascular inflammation by reducing recruitment of inflammatory leukocyte to the brain of infected mice. To test this idea, brain-infiltrating leukocytes were purified from anti-IP-10 treated and control mice at day 6 p.i., stained with fluorescent antibodies and analysed by flow cytometry. The percentage of NK cells, CD4^+^ and CD8^+^ T cells recruited to the brain during infection was similar in anti-IP-10-treated and control mice (Figure 3A). However, the total number of sequestered leukocytes was significantly reduced in anti-IP-10-treated mice compared to controls (control: 226133±40014; anti-IP-10: 94933±13897; p<0.05, Mann-Whitney test). Consistently, the absolute number of CD4^+^ and CD8^+^ T cells was reduced by 50% in anti-IP-10-treated animals compared to controls (Figure 3C–E). No significant differences were found in the number of NK cells in brains of anti-IP-10-treated or control mice (Figure 3B). Analysis of chemokine receptor usage of brain-infiltrating T cells showed that the majority of αβT cells present in brain blood vessels of malaria-infected control mice expressed CXCR3 but not CCR5 (Figure 3F). Similarly, over 70% of αβT cells recovered from brains of anti-IP-10-treated mice expressed this receptor (Figure 3F), suggesting that 8A7 blocking effect of IP-10 may not be absolute or that in the absence of IP-10 other CXCR3 chemokines can recruit leukocytes to the brain during infection.

### Anti-IP-10 treatment does not alter the intrinsic capacity of T cells to migrate in response to chemotactic stimuli

**Figure 4:**
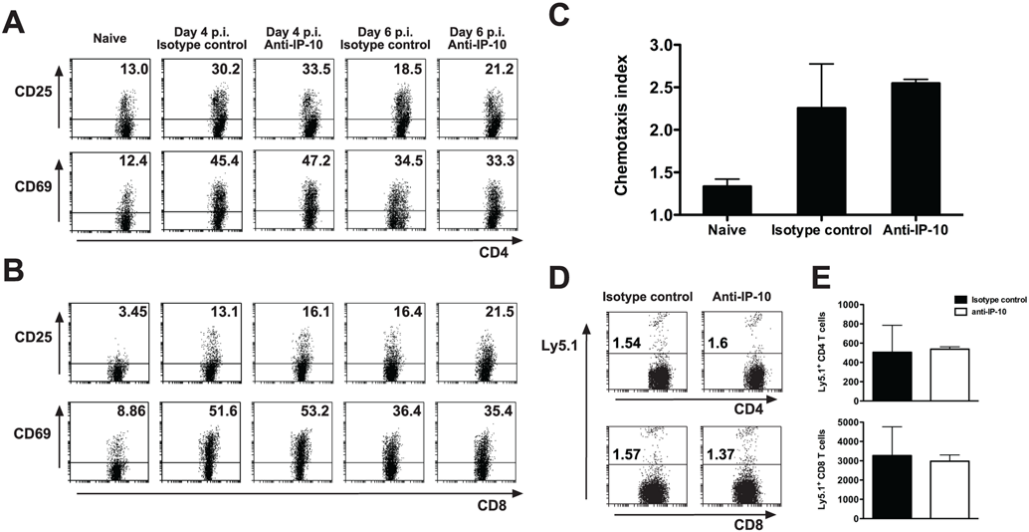
Anti-IP-10 treatment does not intrinsically alter T cell function. C57BL/6 mice were infected with *P.berghei* ANKA and treated with anti-IP-10 or isotype control antibodies. Splenocytes were isolated at different times p.i., stained with fluorescent antibodies and analysed by flow cytometry. The percentage of CD69^+^, CD25^+^ cells was analysed in gated CD4^+^NK1.1^−^ (A) and CD8^+^NK1.1^−^ (B) lymphocytes. (C) T cells were isolated from anti-IP-10 and isotype control-treated mice on day 5 p.i., placed in the upper wells of Transwell inserts and their chemotactic response to IP-10 (100 ng/ml) was determined. Bars represent means of 3 samples±SEM. *P. berghei* ANKA-infected C57BL/6 Ly5.1^+^ mice were treated with anti-IP-10 or isotype control antibodies. Splenic Ly5.1^+^ T cells were isolated on day 6 p.i., adoptively transferred into malaria-infected (day 4 p.i.) C57BL/6 Ly5.2^+^ recipients and 2 days later, brain-infiltrating leukocytes were isolated, stained with fluorescent antibodies and analysed by flow cytometry. The percentage (D) and absolute number (E) of Ly5.1^+^ CD4^+^ and Ly5.1^+^ CD8^+^ cells were determined. Bars represent means of 3 samples±SD.

To investigate whether anti-IP-10 treatment had an effect on T cell activation during malaria, we first examined the percentage of activated CD4^+^ and CD8^+^ T cells expressing CD69 and CD25 in anti-IP-10 and isotype-control-treated mice after *P. berghei* ANKA infection. Figures 4A and B show that the expression of CD25 and CD69 on splenic CD4^+^ and CD8^+^ T cells increased in response to malaria infection. No differences were found in the percentage of activated T cells from anti-IP-10-treated and control mice. In another set of experiments, we sought to determine whether anti-IP-10-treament altered the intrinsic capacity of cells to migrate in response to chemotactic stimuli. To that end, splenic T cells were isolated from anti-IP-10 and isotype-control-treated mice at day 5 p.i. with *P. berghei* ANKA and their ability to migrate in response to IP-10 was evaluated in an *in vitro* chemotaxis assay. As expected, T cells from isotype control-treated malaria-infected mice readily migrated in response to IP-10 compared to cells from naïve mice (Figure 4C). No significant differences were found between the chemotactic response of T cells isolated from control and anti-IP-10-treated mice (Figure 4C). The migratory properties of T cells were also analysed *in vivo*. For that, *P. berghei* ANKA-infected C57BL/6 Ly5.1^+^ mice were treated with anti-IP-10 or isotype control antibodies. Splenic Ly5.1^+^ T cells were isolated on day 6 p.i., adoptively transferred into malaria-infected (day 4 p.i) C57BL/6 Ly5.2^+^ recipients and 2 days later, their ability migrate to the brain in response to infection was evaluated by flow cytometry. Similar percentages (Figure 4D) and absolute numbers (Figure 4E) of brain-infiltrating Ly5.1^+^ CD4^+^ and CD8^+^ T cells were found in mice adoptively transferred with cells from anti-IP-10 and isotype-control treated mice. Taken together, these results indicate that anti-IP-10 treatment does not intrinsically alter the ability of T cells to become activated and to migrate in response to chemokines during malaria-infection. The findings also imply that the reduction in lymphocyte recruitment to inflamed organs observed (Figures 1–3) results from transient neutralization of IP-10 during monoclonal antibody treatment.

### IP-10^−/−^ mice do not develop CM and are markedly resistant to early *P. berghei* ANKA infection

**Figure 5:**
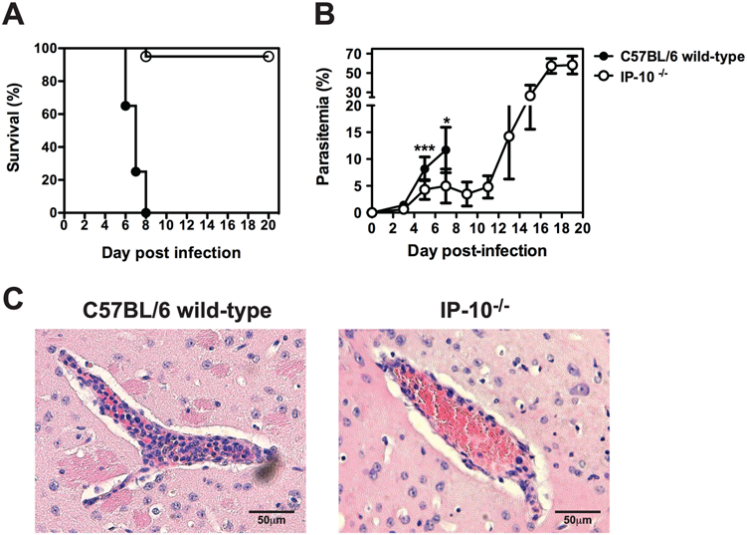
IP-10^−/−^ mice do not develop CM and are markedly resistant to malaria infection. Wild-type and IP-10^−/−^ mice were infected with *P. berghei* ANKA. (A) Survival was monitored daily. (B) Parasitemia was determined by Giemsa-stained blood smears. Each point represents mean±SD, ***p<0.0001, *p<0.05 (Mann-Whitney test). Pooled data from 2 infections (n = 20) is shown. (C) Histological examination of brains from *P. berghei*-infected wild-type and IP-10^−/−^ mice.

**Figure 6:**
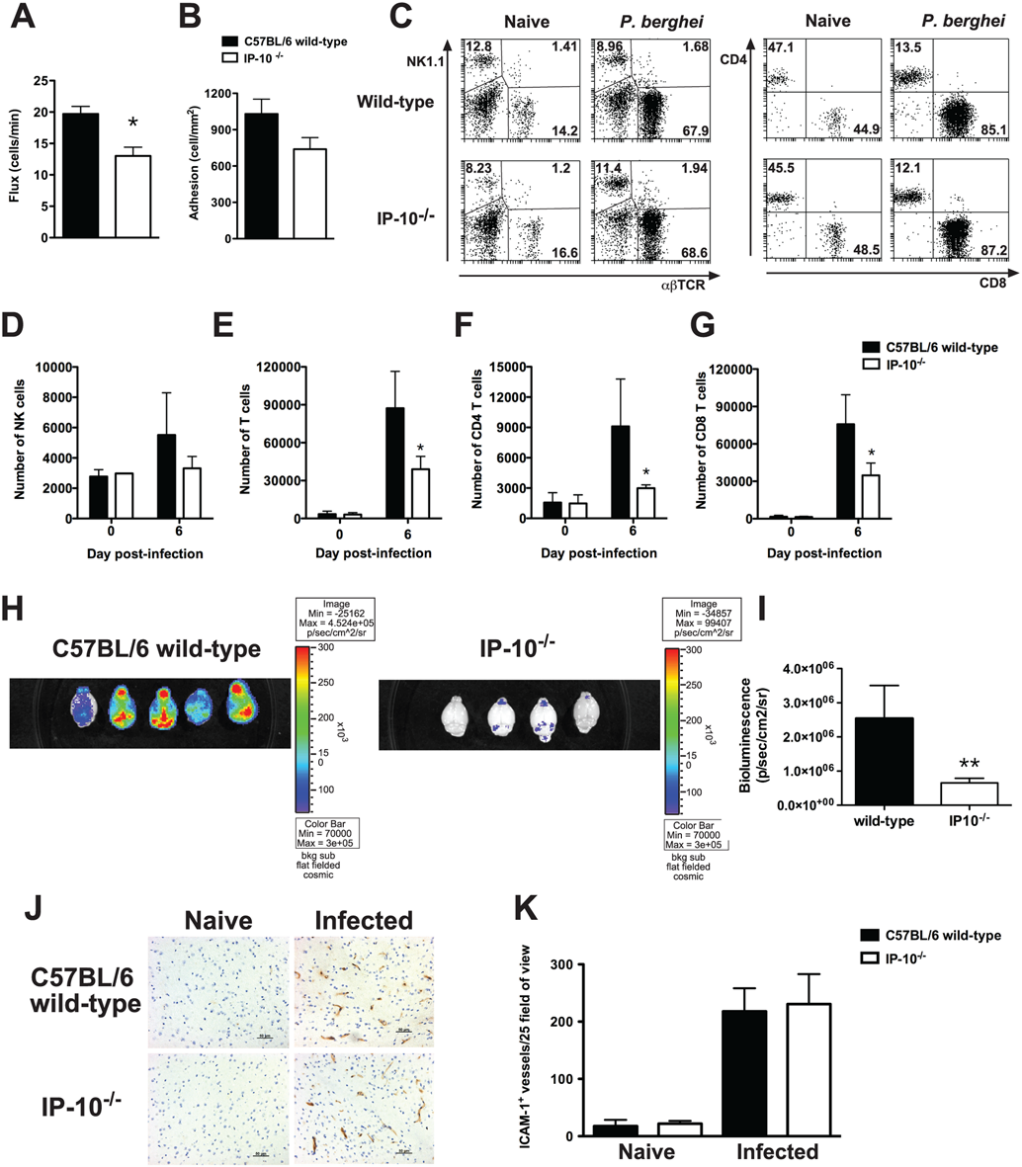
Intravascular infiltration and parasite sequestration are reduced in brains of malaria-infected IP-10^−/−^ mice. Numbers of rolling (A) and adherent (B) cells in brain blood vessels of wild-type and IP-10^−/−^ mice were determined by intravital microscopy on day 5 p.i. with *P. berghei* ANKA. Bars represent means of 4–6 mice±SEM, *p<0.05 (Mann-Whitney test). Brain-infiltrating leukocytes were isolated on day 6 p.i., stained with fluorescent antibodies and analysed by flow cytometry. Percentage (C) and total number of NK cells (D), total T cells (E), CD4^+^ (F) and CD8^+^ T cells (G) were calculated. Bars represent means of 3 samples±SD, *p<0.05 (Mann-Whitney test). Wild-type and IP-10^−/−^ mice were infected with luciferase-expressing *P. berghei* ANKA. (H) Brain-sequestered parasites were visualized 1 h after luciferin injection. (I) Parasite-associated bioluminescence was recorded. Bars represent means of 4–5 samples±SD, **p<0.01. (J) ICAM-1 staining was performed on brain sections of wild-type and IP-10^−/−^ mice prepared on day 6 p.i. (K) The number of ICAM-1 positive vessels was determined. Bars represent means of 4 samples±SD.

IP-10^−/−^ mice have been shown to be partially resistant to *P. berghei*-mediated CM [20]. Our results indicate that specific neutralization with anti-IP-10 antibodies confers high protection levels against cerebral disease. Therefore, we sought to investigate the reasons for this discrepancy. In a first series of experiments, IP-10^−/−^ and C57BL/6 wild-type mice were infected with 1×10^6^ *P. berghei* ANKA pRBC and susceptibility rates to CM and parasitemia levels were determined. All wild-type control animals succumbed to CM between day 6–8 p.i. (Figure 5A). Under these conditions, 95% of IP-10^−/−^ mice did not develop CM and survived into the third week of the challenge (p<0.0001). Parasitemia levels were markedly lower in IP-10^−/−^ compared to wild-type mice in the first week of infection (Figure 5B). This reduction in parasite burdens was more evident than that observed in anti-IP-10-treated mice (Figure 1C). Parasitemia levels of IP-10^−/−^ mice rose only after day 13 p.i.. No signs of cerebral disease were observed in IP-10^−/−^ animals at these late stages of the experimental challenge. Histological examination of brain sections displaying high levels of vascular occlusion in 65–70% of the blood vessels examined confirmed the diagnoses of CM in wild-type mice (Figure 5C). In contrast, IP-10^−/−^ mice showed very reduced vascular occlusion in only 30–40% of blood vessels examined. Real time examination of brain blood vessels by intravital microscopy revealed a significant reduction in the number of rolling cells (Figure 6A) and a trend to reduced number of adherent cells in IP-10^−/−^ compared to wild-type mice (Figure 6B). The composition of brain-sequestered leukocytes was also analysed in malaria-infected IP-10^−/−^ mice by flow cytometry. Although no differences were found between the percentage of NK cells and T cells in brains of IP-10^−/−^ and wild-type mice (Figure 6C), the total number of sequestered leukocytes (wild-type: 239865±40628; IP-10^−/−^: 98133±7087; p<0.05, Mann-Whitney test) as well as CD4^+^ and CD8^+^ T cells recovered from brains of IP-10^−/−^ mice was significantly lower than in wild-type animals (Figure 6D–G). To determine whether the reduced parasite growth rates observed in IP-10^−/−^ mice had an impact on pRBC sequestration, wild-type and IP-10^−/−^ mice were infected with luciferase-expressing *P. berghei* parasites and bioluminescence in the brain was determined as described in Figure 2. Unlike in anti-IP-10-treated mice (Figure 2), parasite-associated bioluminescence was reduced by 80% in brains of IP-10^−/−^ mice compared to wild-type controls (Figure 6H,I). Since in human malaria cytoadhesion of infected erythrocytes to the vascular endothelium is known to be mediated by ICAM-1 [29], the expression of this adhesion molecule was examined in brain blood vessels of *P. berghei*-infected wild-type and IP-10^−/−^ mice by immunohistochemistry (Figure 6J). No significant differences were found in the number of blood vessels expressing ICAM-1 between IP-10^−/−^ mice and wild-type controls (Figure 6K). Taken together, these results indicate that genetic deletion of IP-10 protects from CM by reducing recruitment of inflammatory leukocytes and by preventing pRBC sequestration in the brain.

### Lack of IP-10 enhances splenic anti-*P. berghei* immune responses

**Figure 7:**
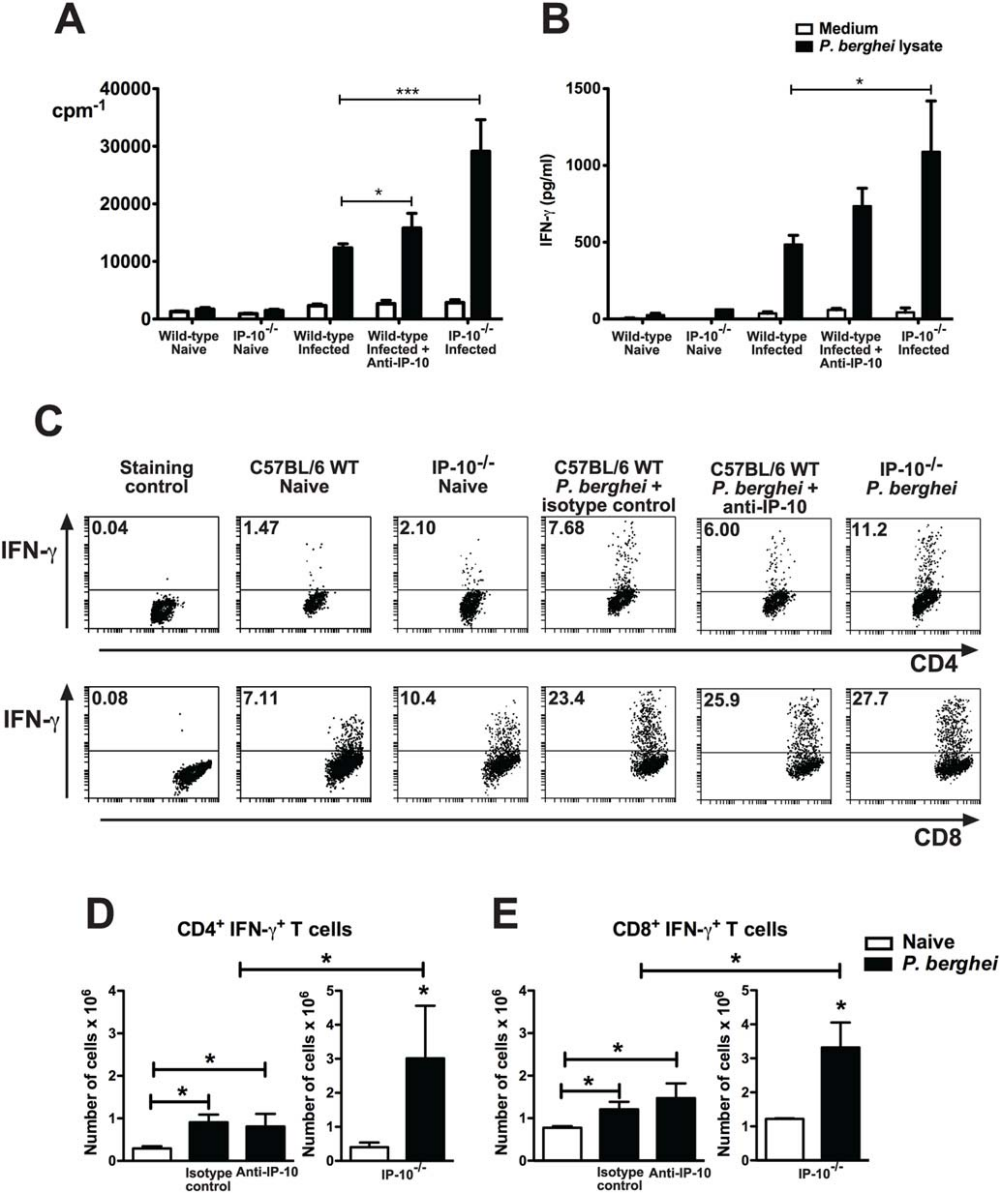
Lack of IP-10 enhances splenic immune responses to malaria. CD4^+^ T cells from IP-10^−/−^, anti-IP-10-treated and control mice (n = 6) were isolated on day 5 p.i. with *P. berghei* ANKA and stimulated *in vitro* with parasite lysate. (A) Proliferation was measured by [^3^H]-thymidine incorporation and (B) IFN-γ levels in culture supernatants were determined by ELISA. Bars represent means±SEM, *p<0.05, ***p<0.005 (Student t-test). Splenocytes from malaria-infected mice were stained with anti-CD4, anti-CD8 and anti-IFN-γ antibodies and analysed by flow cytometry. Percentage (C) and absolute number of IFN-γ^+^ CD4^+^ (D) and CD8^+^ (E) T cells were determined. Each experiment is representative of 2 infections. Representative dot plots are shown. Bars represent means of 3 mice±SD, *p<0.05 (Mann-Whitney test).

The fact that lack of IP-10 during malaria reduced parasite burden suggested that the absence of this chemokine has a beneficial effect for the development of parasite-specific responses involved in the control of infection. Interestingly, in addition to its chemotactic activity, IP-10 has been shown to stimulate and not inhibit the induction of several effector responses. To address this paradox, the induction of immune responses to malaria was investigated in anti-IP-10-treated and IP-10^−/−^ mice. Since the spleen is a key organ involved in the initiation of immune responses to blood-stage malaria [30], splenic parasite-specific responses were examined. In a first set of experiments, CD4^+^ T cells from anti-IP-10-treated, IP-10^−/−^ and control mice were isolated at day 5 p.i. and proliferative responses to parasite lysate as well as IFN-γ and IL-4 production were determined. A small but significant increase in parasite-specific proliferation was observed in CD4^+^ T cells of anti-IP-10-treated animals compared to controls (Figure 7A). These responses as well as IFN-γ production were significantly more pronounced in CD4^+^ T cells from IP-10^−/−^ mice (Figure 7A,B). IL-4 production was virtually absent in T cells from malaria-infected C57BL/6 mice (not shown). No significant differences were found in proliferative responses or cytokine production to anti-CD3 antibody across experimental groups (not shown). The frequency and absolute number of IFN-γ producing cells was also evaluated in gated CD4^+^ and CD8^+^ T cells by flow cytometry. Similar frequencies of IFN-γ^+^ CD4^+^ and CD8^+^ cells were found among splenocytes of malaria-infected anti-IP-10-treated and control mice (Figure 7C). These percentages were somewhat higher in IP-10^−/−^ mice. A small increase in the absolute number of IFN-γ-producing CD4^+^ and CD8^+^ T cells was found in anti-IP-10-treated compared to naïve mice (Figure 7D,E). Like proliferation, genetic deletion of IP-10 resulted in a more dramatic effect, as the total number of CD4^+^ and CD8^+^ IFN-γ-secreting cells in IP-10^−/−^ mice was around 3 times higher than in wild-type animals (Figure 7D,E). To examine whether IP-10 deletion also affected systemic IFN-γ responses to malaria, serum IFN-γ levels were determined in wild-type and IP-10^−/−^ mice at day 5 p.i. Interestingly, serum IFN-γ content was significantly lower in malaria-infected IP-10^−/−^ mice compared to wild-type control animals (Figure S1). Together these results suggest that lack of IP-10 enhances splenic but not systemic immune responses to malaria.

### Inhibition of IP-10-mediated trafficking favors retention of parasite-specific T cells in spleens of malaria-infected mice

**Figure 8:**
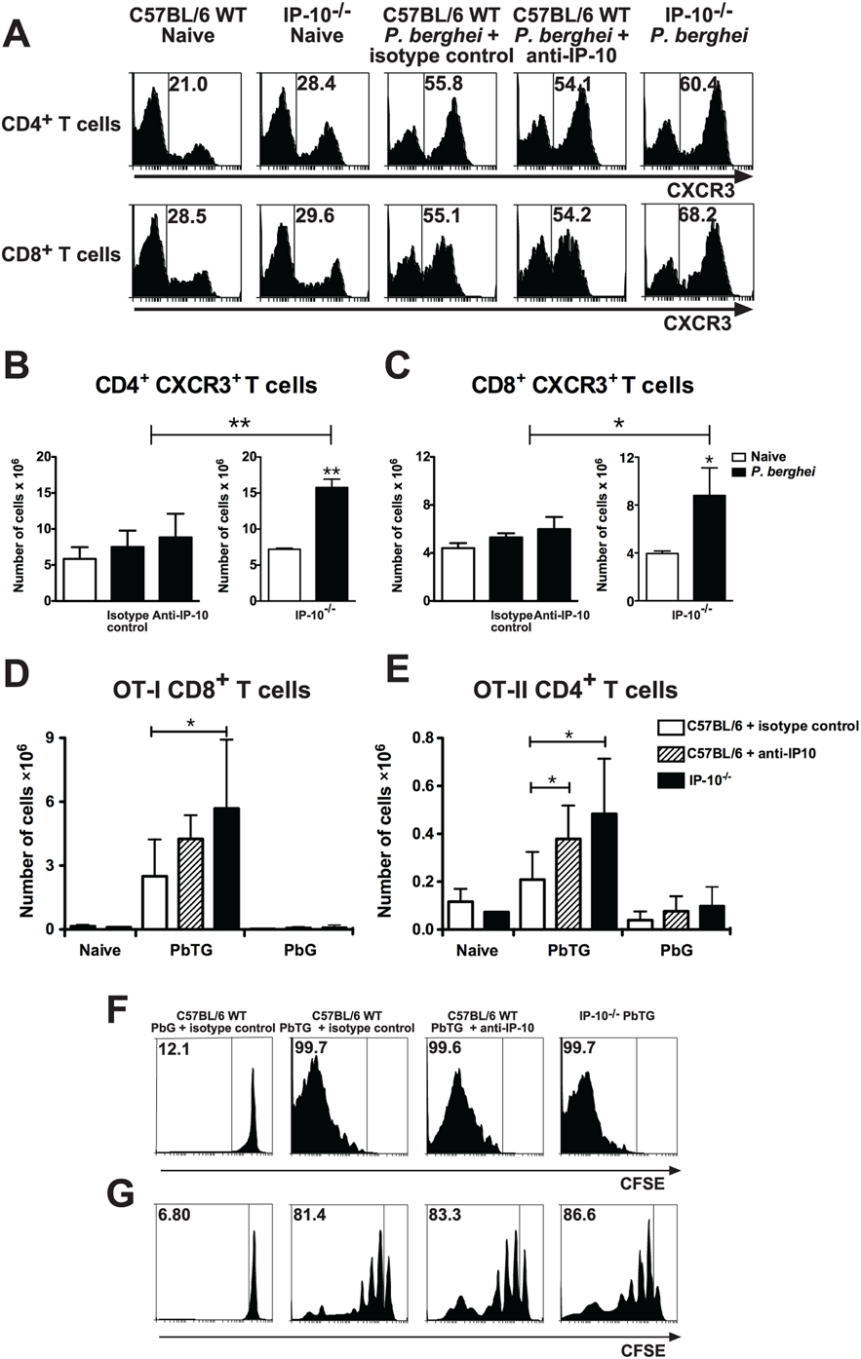
Inhibition of IP-10-mediated trafficking favors splenic accumulation of parasite-specific T cells. Splenocytes from malaria-infected IP-10^−/−^, anti-IP-10-treated or control mice were purified and stained with anti-CD4, anti-CD8 and anti-CXCR3 antibodies for analysis by flow cytometry. Percentage (A) and absolute number of CXCR3^+^CD4^+^ (B) and CD8^+^ (C) T cells were calculated. Bars represent means of 3 mice±SD, *p<0.05, **p<0.01 (Mann-Whitney test). Each experiment is representative of 3 infections. Wild-type and IP-10^−/−^ mice were adoptively transferred with OT-I CD8^+^ and OT-II CD4^+^ T cells (Ly5.1^+^) 2 days before challenge with OVA-expressing PbTG or control PbG parasites. Wild-type mice were treated with anti-IP-10 or isotype control antibodies. On day 5 p.i., splenocytes were stained with anti-CD4, anti-CD8 and anti-Ly5.1 antibodies and analysed by flow cytometry. Absolute numbers of OT-I CD8^+^ (D) and OT-II CD4^+^ T (E) cells were determined. Bars represent means of 5–9 samples±SD, *p<0.05 (Mann-Whitney test). CFSE-labelled CD8^+^ and CD4^+^ T cells from Ly5.1^+^ OT-I and OT-II mice respectively, were adoptively transferred into Ly5.2^+^ recipients. Two days later, mice were infected with PbTG or PbG parasites. Splenocytes were harvested on day 5 p.i. and CFSE staining was assessed on gated Ly5.1^+^ CD8^+^ (F) or Ly5.1^+^ CD4^+^ (G) cells by flow cytometry. Representative histograms are shown.

It has been proposed that the spleen is the source of activated inflammatory cells that migrate to the site of parasite sequestration in target organs during severe malaria [31]. In that context, we postulated that the increased number of splenic IFN-γ-producing T cells observed during malaria when IP-10 mediated chemotaxis was impaired reflected a retention of CXCR3^+^ T cells in spleens of infected animals. To address this hypothesis, we first examined the expression levels and total numbers of CXCR3^+^ T cells in spleens of anti-IP-10-treated, IP-10^−/−^ and control animals at day 5 p.i. with *P. berghei* ANKA. Both CD4^+^ and CD8^+^ T cells from C57BL/6 mice up-regulated CXCR3 expression levels during infection (Figure 8A). This response was of similar magnitude in anti-IP-10-treated animals and it was somewhat higher in IP-10^−/−^ mice (Figure 8A). In spite of no evident splenomegaly, a 2-3-fold increase in the number of CXCR3^+^ CD4^+^ and CD8^+^ T cells was observed in IP-10^−/−^ mice compared to controls (Figure 8B,C). Thus, in the absence of IP-10-dependent trafficking, CXCR3^+^ T cells accumulate in spleens of malaria-infected animals.

To determine whether parasite-specific T cells were retained in the spleen when IP-10 is impaired, we used transgenic parasites expressing green fluorescent protein (GFP) alone (PbG) or fused to T cell epitopes including the MHC I and MHC II-restricted epitopes from OVA (PbTG) [17]. These parasites allowed us to track the response of T cells specific for parasite-expressed OVA by using transgenic T cells from OT-I and OT-II mice. These transgenic T cells recognise the encoded MHC I and MHC II-restricted OVA epitopes, respectively. To enumerate specific T cells during infection, OT-I or OT-II cells from Ly5.1^+^ mice were transferred into congenic C57BL/6 (Ly5.2^+^) mice 2 days before infection with PbTG or PbG, and transgenic T cells numbers were enumerated in the spleen on day 5 p.i. (Figure 8D,E). The number of OT-I and OT-II cells increased in spleens of PbTG-infected mice relative to naïve and PbG-infected mice, indicating parasite-specific T cell proliferation in response to infection. Importantly, the number of both OT-I (Figure 8D) and OT-II (Figure 8E) cells were significantly increased in IP-10^−/−^ mice relative to wild-type controls, a finding that was reflected in anti-IP-10 treated mice, though only reaching significance for OT-II cells (Figure 8E). The increase in specific T cell numbers in IP-10^−/−^ mice was not due to increased proliferation of these T cells, since transfer of CFSE-labelled transgenic OT-I and OT-II cells under similar conditions revealed identical proliferative profiles in wild-type, anti-IP-10 treated and IP-10^−/−^ mice (Figure 8F,G). These results indicate that inhibition of IP-10-mediated trafficking in malaria favors retention of parasite-specific T cells in the spleens of infected mice. These results also suggest that splenic accumulation of parasite-specific T cells might be responsible for the reduced parasitemia levels observed when IP-10-mediated chemotaxis is impaired (Figures 1C and 5B).

**Figure 9:**
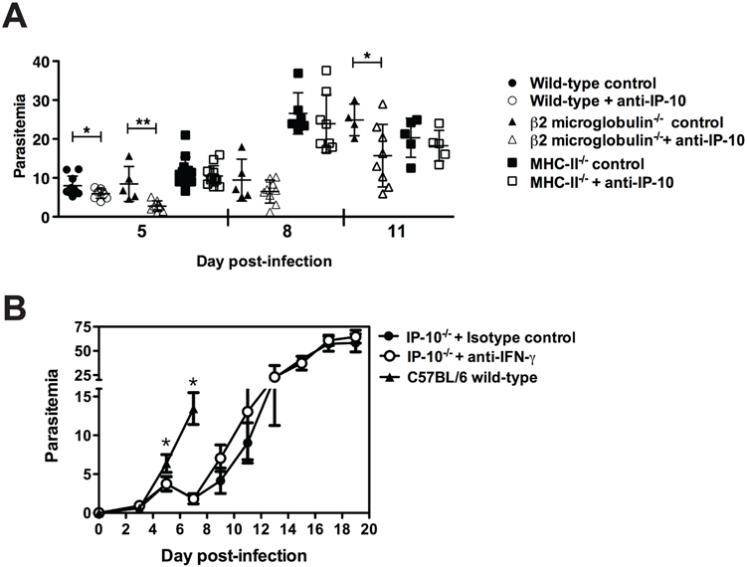
CD4^+^ T cells contribute to the control of parasite burden. (A) Wild-type, β_2_-microglobulin^−/−^ and MHC-II^−/−^ mice (n = 5–10) were infected with *P. berghei* ANKA and then treated with anti-IP-10 or isotype control antibodies. Parasitemia was determined at different days p.i.. Scatter plots represent mean parasitemia±SD, *p<0.05, **p<0.01 (Mann-Whitney test). (B) IP-10^−/−^ mice were infected with *P. berghei* ANKA. Mice were treated with anti-IFN-γ or isotype control antibodies every second day starting on day 1 p.i. Untreated C57BL/6 mice were included. Each point represents mean parasitemia±SD, *p<0.05 between C57BL/6 and both anti-IFN-γ or isotype control-treated IP-10^−/−^ mice, (Mann Whitney test).

To support this view and to determine whether a particular T cell subset was required for the improved control of parasite burden that takes place when IP-10-mediated chemotaxis is prevented, we investigated the effect that trafficking inhibition has on parasitemia levels of mice lacking CD4^+^ or CD8^+^ T cells. To that end, *P. berghei* ANKA infected C57BL/6 wild-type, MHC II^−/−^ and β_2_-microglobulin^−/−^ mice were injected with anti-IP-10 or isotype control antibodies, and parasitemia levels were determined at different times p.i. Similar to wild-type mice, anti-IP-10-treatment significantly reduced parasitemia in β_2_-microglobulin^−/−^ mice (Figure 9A). In contrast, IP-10 neutralization did not facilitate control of parasitemia in infected MHC II^−/−^ mice. Thus these results suggest that CD4^+^ T cell-mediated responses are required for the increased control of parasite burdens that occurs during malaria infection when IP-10-meditated chemotaxis is inhibited.

Figure 7 indicates that genetic deletion of IP-10 (and to lesser extent anti-IP-10 treatment) results in retention of IFN-γ^+^ cells in the spleen of malaria-infected animals. To examine whether the increased frequency of IFN-γ producing cells contributes to the efficient control of parasitemia occurring in the absence of IP-10-mediated trafficking, IP-10^−/−^ mice were infected with *P. berghei* ANKA, treated with neutralizing anti-IFN-γ antibodies or an isotype control and parasitemia levels were determined. Parasitemia levels of C57BL/6 wild-type control mice were significantly higher than those from both anti-IFN-γ or istoype control-treated IP-10^−/−^ mice on days 5 and 7 p.i. (Figure 9B). IFN-γ did not appear to play a major role in the control of parasite burden in this model as neutralization with antibodies over the course of infection did not significantly change parasitemia levels of IP-10^−/−^ mice compared to isotype control-treated animals.

## Discussion

This study provides evidence that IP-10 has a critical role in the development of CM pathogenesis. Neutralization with specific antibodies or genetic deletion in IP-10^−/−^ mice protected malaria-infected mice from cerebral disease and fatalities although by non-identical mechanisms. Passive transfer of anti-IP-10 antibodies did not intrinsically alter lymphocyte activation or function and thus protected from CM mainly by reducing recruitment of inflammatory leukocytes to brain blood vessels, whereas genetic deletion of IP-10 in knockout mice not only alleviated intravascular inflammation but also reduced pRBC sequestration in the brain and peripheral parasitemia. The increased resistance to infection observed in the absence of IP-10-mediated trafficking was associated with retention of parasite-specific T cells in the spleen. Thus our results support the notion that disruption of IP-10-dependent trafficking not only reduces recruitment of pathogenic cells to target sites but also facilitates control of parasite burden by favoring accumulation of effector cells in secondary lymphoid organs.

IP-10 has been shown to play an important role in lymphocyte recruitment in several inflammatory conditions. Similar to our results, antibody neutralization studies reported decreased T cell recruitment into the CNS in experimental autoimmune encephalomyelitis [32] and during infection with the neurotropic mouse hepatitis virus (MHV) [33]. In the present study, antibody treatment as well as infection of IP-10^−/−^ mice resulted in a partial reduction in leukocyte recruitment to the brain, which was sufficient to alleviate disease and prevent fatalities. Interestingly, the majority of brain-sequestered T cells found in anti-IP-10-treated mice expressed CXCR3, suggesting that in the absence of IP-10 other CXCR3 ligands are able to mediate trafficking to the brain during infection but at levels that are not sufficient to induce severe disease. A role for MIG in this process cannot be excluded since high levels of this chemokine have been found in brains of malaria-infected animals [19],[21] and MIG^−/−^ mice were reported to be partially resistant to *P. berghei* ANKA infection [20].

In contrast to the high protection levels against CM in IP-10^−/−^ mice detected here, a previous study [20], in which mice were challenged with higher parasite doses resulted in 60% protection from disease. In general, *P. berghei* ANKA infection results in consistent CM induction when mice are infected with doses ranging from 1×10^5^–1×10^6^ pRBC. It is possible that even in CM-susceptible animals, infection with higher parasitic inocula may accelerate parasite growth rates resulting in higher parasitemia and concomitant haemolytic anemia (unpublished observations). Under these conditions, fatalities may arise from the combination of different overlapping disease syndromes. Thus inconsistencies among studies might reflect the development of other malaria-associated syndromes contributing to the increased fatality rates of IP-10^−/−^ mice inoculated with higher parasite doses. However, with the current evidence these differences remain difficult to interpret, as neither parasitemia nor other pathological endpoints in IP-10^−/−^ mice were previously investigated.

In addition to its chemotactic activity, IP-10 has been shown to participate in the induction of immune responses. Therefore, we initially reasoned that lack of IP-10 during malaria could inhibit the induction of inflammatory lymphocytes that then migrate to target organs. Instead, we found that despite similar *in vivo* proliferation rates, there were increased numbers of parasite-specific T cells in spleens of anti-IP-10-treated and IP-10^−/−^ mice after malaria challenge compared to controls, indicating that trafficking inhibition resulted in retention of CXCR3^+^ T cells in this organ. These effects were more evident in the knockout animals, where frequencies of IFN-γ producing cells were also increased, suggesting that splenic T cell retention facilitates further activation and expansion. IP-10 blockade has been shown to inhibit the induction of splenic effector immune responses in tumor models [34], MHV [23] and *Toxoplasma gondii* infection [35]. In the latter study, IP-10 neutralization was shown to inhibit the influx (and subsequent expansion) of antigen-specific CD4^+^ and CD8^+^ T cells into infected-spleens, which resulted in impaired parasite clearance. Presumably, these cells entered the bloodstream after activation in lymph nodes and found their way into the inflamed tissue. Unlike *T. gondii*, malaria asexual stages are blood-borne parasites. Thus the spleen, which is a crucial organ involved in blood filtration constitutes a key site in the initiation of immune responses to the parasite. The importance of the spleen in the immunity to malaria is highlighted by the fact that splenectomy impairs parasite clearance in both humans [36] and mice [37],[38]. Moreover, it has been suggested that the spleen is the organ of initial induction of inflammatory cells that then migrate to the site of parasite sequestration in target organs, as splenectomized mice do not develop *P. berghei*-mediated CM [39]. Our results here are consistent with such hypothesis and suggest that the effect that IP-10 has on the induction of adaptive responses varies depending on the nature of the microbial stimulus, pathogen tropisms and the tissue of origin of the acquired response to infection.

Although lack of IP-10 increased frequencies of splenic IFN-γ producing T cells, serum levels of this cytokine during malaria infection were not increased but reduced in IP-10 deficient animals compared to wild-type controls. Further work is required to confirm whether the low IFN-γ content in sera reflects reduced frequencies of circulating cells secreting this cytokine as IFN-γ^+^ CXCR3^+^ T cells appear to accumulate in responding lymphoid organs when IP-10-mediated trafficking is inhibited.

CD4^+^ T cells have been shown to play a central role in the development of protective immunity against blood stage malaria both in humans [40] and rodent models [41]. They produce cytokines that enhance macrophage phagocytic activity and are essential to provide help for antibody production. Consistently, our findings indicate that the enhanced control of parasite burden that occurs when IP-10-mediated trafficking is impaired requires CD4^+^ T cells. Interestingly, experimental evidence indicates the same T_H_1 responses that contribute to the control of infection can induce severe disease and pathology [6]. Our resultsconfirm and extend these observations demonstrating that 1) modulating the homing of cellular immune responses to the spleen is critical for reaching a balance between protective immunity and pathogenesis and 2) inflammatory processes that occur during infection are not only detrimental for their involvement in severe disease but can also compromise the induction of anti-parasite immunity by inducing T cell migration away from the spleen.

The precise mechanism by which CD4^+^ T cells accumulating in the spleen in the absence of IP-10-mediated trafficking control parasite burden needs to be further investigated. Although these cells appeared to produce IFN-γ, *in vivo* neutralization of this cytokine did not limit the ability of IP-10^−/−^ mice to efficiently control parasitemia. Our findings here are in agreement with previous observations [9] in CBA/Ca mice, supporting the notion that IFN-γ does not play a major role in the control of parasite growth in the *P. berghei* ANKA rodent model of CM. It remains to be determined whether other T_H_1 cytokines such as TNF-α contribute to the splenic control of parasitemia in IP-10 deficient mice as it has been observed in previous investigations using *P. berghei* ANKA parasites [6].

Throughout this study, splenic T cell accumulation and the associated control of parasitemia was more evident in IP-10^−/−^ mice than in anti-IP-10 treated animals. Although it is reasonable to postulate that the inhibition of pRBC sequestration in IP-10^−/−^ mice reflects high levels of resistance to infection observed in these animals, we reasoned that it was also possible that constitutive lack of IP-10 could alter the adhesive properties of the brain microvasculature, resulting in reduced pRBC cytoadhesion. In fact, IP-10^−/−^ mice have impaired IFN-γ production in the brain during MHV infection [23] and this cytokine is known to up-regulate expression levels of adhesion molecules such as ICAM-1 and VCAM-1 in brain endothelial cells [42]. In the current study, although genetic deletion of IP-10 substantially reduced systemic IFN-γ levels, it did not affect ICAM-1 expression on the brain microvasculature during infection. However, whether reduced expression of other adhesins inhibits sequestration of *P. berghei* pRBC is still unclear as the precise mechanisms responsible for cytoadhesion in rodent malaria have not been established.

Recent studies have identified IP-10 as a biomarker associated with increased risk of *P. falciparum*-mediated CM mortality [25],[26]. In addition to pRBC sequestration, emerging data has provided evidence for intravascular leukocyte infiltration in human CM, implying its association with disease induction [15],[16],[43]. Further work is required to determine whether these inflammatory cells are recruited to the brain via an IP-10-dependent mechanism as it occurs in mice. Nevertheless, this study shows that it is possible to prevent fatalities by administration of anti-IP-10 antibodies during the course of infection, providing proof of concept for the therapeutic potential of anti-leukocyte trafficking strategies as adjunctive therapy to improve treatment outcomes of CM.

## Materials and Methods

### Ethics statement

All experiments carried out were approved by the Walter and Eliza Hall Institute of Medical Research Animal Ethics Committee and in compliance with the Committee's requirements.

### Mice and infections

Eight to 12 week-old C57BL/6, IA-β^−/−^ (F_10_ generation) [44], β2-microglobulin^−/−^ (F_11_ generation) or IP-10^−/−^ (F_9_ generation) mice (The Jackson laboratory, ME) were used. A polyclonal line of *P. berghei* ANKA was used in the study. Parasites were maintained as stabilates in liquid nitrogen. Groups of 10–15 mice were injected intraperitoneally (i.p.) with 1×10^6^ freshly passaged *P. berghei* ANKA pRBC. In some experiments, mice were injected intravenously (i.v.) with 200 µg of anti-IP-10 monoclonal antibodies or an isotype control from day 3–9 p.i. or from day 5–9 p.i. Alternatively, mice were injected i.p. with 0.5 mg of anti-IFN-γ (clone HB170) or an isotype control every second day starting on day 1 p.i. Parasitemia was assessed by counting 10 microscope fields from Giemsa-stained smears of tail blood prepared every 2–3 days. Mortality was checked daily. Mice were judged as developing CM if they displayed neurological signs such as ataxia, loss of reflex and hemiplegia, and died between days 6 to 10 p.i. with low parasitemia. All experiments complied with the Walter & Eliza Hall Institute Animal Ethics Committee requirements.

### Production of anti-IP-10 monoclonal antibodies

Wistar rats were immunized with recombinant mouse IP-10 in complete Freund's adjuvant and then boosted twice with antigen in incomplete Freund's adjuvant. Splenocytes from immunized rats were fused to P3X63 myeloma cells lines using standard polyethylene glycol 150 protocols. Positive clones were detected by ELISA and then subcloned twice by limiting dilution. Selected clones were cultured in Hybridoma Serum Free Medium using Cellmax/Miniperm Bioreactors. Monoclonal antibodies were then purified by Protein A chromatography.

### Chemotaxis assays

T cells were purified from splenocytes of malaria-infected mice with a negative isolation kit (Invitrogen Dynal, Oslo, Norway) following the manufacturer's instructions. For migration studies Transwell inserts (Corning Costar, Acton, MA) containing T cells (5×10^5^/100 µl) were placed in duplicates or triplicates in 24-well plate wells with mouse recombinant IP-10, MIG (200 ng/ml) or I-TAC (400 ng/ml) (all chemokines from Peprotech, Rocky Hill, NJ). In some experiments, anti-IP-10 antibodies were added to the wells and incubated for 30 min before addition of cells. The cells were incubated for 4 h at 37°C with 5% CO_2_. Cells in the lower chambers were collected and counted in a Neubauer hemocytometer. A chemotaxis index was calculated by dividing the number of cells migrating in response to chemokines by the number of cells migrating in wells with medium alone.

### Brain histology

For histological analysis of cerebral pathology, brains from *P. berghei*-infected mice were taken into 10% neutral-buffered formalin, sectioned (5 µm) and stained with Haematoxylin/Eosin. Slides were coded and scored blind for histological evidence of cerebral syndrome.

### Intravital microscopy

Mice were prepared for cerebral intravital microscopy exactly as described previously [45]. Briefly, animals were anaesthetized by i.p. injection of 150 mg/kg ketamine hydrochloride (Caringbah, NSW, Australia) and 10 mg/kg xylazine (Bayer Pharmaceuticals, Pymble, NSW, Australia) and maintained at 37°C using a heating pad. The animal's head was held in a stereotaxic board and the skull was exposed by a skin incision and the periosteum over the parietal bone was removed. A craniotomy was performed in the right parietal bone and a stainless steel superfusion chamber was applied to the skull with bone wax and Loctite 406 rapid adhesive (Loctite Australia, Caringbah, NSW, Australia). The chamber was filled with artificial CSF then the bone cap and the underlying dural membrane were removed to expose the pial vessels. The chamber was then sealed using a coverslip held in place with vacuum grease and CSF infusion maintained at 0.3 ml/min. The pial microvasculature was observed using an intravital microscope (Axioplan 2 Imaging; Carl Zeiss, Australia) with a ×40 water immersion objective lens (Achroplan X40/0.80 NA, Carl Zeiss). Images were visualized using a SIT video camera (Dage-MTI VE-1000; Sci Tech Pty. Ltd.). Leukocytes were detected by i.v. injection of 50 µl of 0.05% rhodamine 6G (Sigma Co, Australia). Three-five postcapillary venules (25–50 µm in diameter) were examined and average data generated for each animal. Leukocyte rolling flux and adhesion were calculated as previously described [45].

### *In Vivo* bioluminescence imaging

Mice were infected (1×10^5^ pRBC, i.v.) with a transgenic *P. berghei* ANKA line expressing luciferase and GFP under the control of the elongation factor 1-α promoter [28]. On day 6 p.i, mice were sacrificed and brains were removed after perfusion. Luciferase-expressing pRBCs were visualized in the brain with an I-CCD photon-counting video camera and *in vivo* imaging system (IVIS 100; Xenogen, Alameda, CA). Bioluminescence generated by luciferase transgenic parasites in brain tissue was measured according to the manufacturer's instructions using the same regions of measurement for all samples being compared.

### Purification and analysis of brain-sequestered leukocytes

Brain-sequestered leukocytes were purified on day 6 p.i with *P. berghei* ANKA as described before [14]. Briefly, euthanized mice were perfused to remove circulating leukocytes. Brains were then removed, crushed in RPMI medium and pushed through a cell mesh. The tissue extract was centrifuged at 200×g for 10 min and the pellet was dissolved in RPMI containing 0.05% Collagenase D (Worthington, Lakewood, NJ) and 2 U/ml DNAase I (Sigma). After 1 h incubation at 22°C, the mixture was filtered through a cell strainer, seeded on a 35% Percoll (Amersham Bioscience, Uppsala, Sweden) cushion and centrifuged at 400×g for 20 min at 22°C. The pellet was collected and erythrocytes were lysed with Tris-NH_4_Cl Buffer. After washing, recovered cells were incubated with anti-CD16 antibody, washed and stained with PE-anti-NK1.1 (PK136), APC-anti-TCR (H57-597), FITC-anti-CD4 (L3T4) and PerCP-Cy5.5-anti-CD8 (53-6.7). Alternatively, cells were stained with FITC-anti-NK1.1, APC-anti-TCR, PE-anti-CXCR3 (220803) (R&D Systems) and biotinylated anti-CCR5 (C34-3448). The cells were then washed and incubated with a Streptavidin-PerCP-Cy5.5 conjugate (all antibodies from BD Pharmingen, San Diego, CA, except otherwise indicated). After washing, cells were resuspended in PBS and analysed by flow cytometry.

### Adoptive transfer of Ly5.1^+^ lymphocytes

C57BL/6 Ly5.1^+^ mice were infected with *P. berghei*-ANKA (1×10^6^ pRBC i.p.) and then treated with anti-IP-10 or isotype control antibodies. Mice were euthanized on day 6 p.i. and splenic T cells were purified with a negative isolation kit (Invitrogen Dynal, Oslo, Norway) following the manufacturer's instructions. Ly5.1^+^ T cells were then adoptively transferred into malaria-infected (day 4 p.i.) C57BL/6 Ly5.2^+^ recipient animals. Two days later, brains were harvested and infiltrating leukocytes were incubated with anti-CD16 antibody, washed and stained with PE-anti-Ly5.1, PerCPCy5.5-anti-CD8 and APC-anti-CD4 for detection of adoptively transferred T cells. After washing, cells were resuspended in PBS and analysed by flow cytometry.

### Flow cytometry

Spleen cells were incubated with anti-CD16 antibody, washed and then stained with FITC-anti-NK1, APC-anti-CD4, PerCP-Cy5.5-anti-CD8 and PE-anti-CXCR3 for 1 h on ice. For analysis of T cell activation, splenocytes were stained with APC-anti-CD4 or PerCP-Cy5.5-anti-CD8 together with PE-anti-NK1.1 and either FITC-anti-CD69 (H1.2F3) or FITC-anti-CD25 (7D4). For intracellular cytokine staining experiments, splenocytes were incubated with PE-anti-NK1.1, APC- anti-CD4 or PerCP-Cy5.5- anti-CD8 for 1 h. After washing, cells were fixed and permeabilized with Citofix/Citoperm (BD Pharmingen, San Diego, CA), and incubated with either FITC-anti-IFN-γ or an isotype-matched antibody. The cells were then washed twice, resuspended in PBS and analysed in a FACSCalibur cytofluorometer (BD Biosciences, NJ). Viable cells were gated by forward and side scatter.

### Immunohistochemistry

ICAM-1 staining was conducted on acetone-fixed brain sections as described before [46]. Briefly, anti-ICAM antibodies were detected with appropriate secondary detection reagents and horseradish peroxidase according to the manufacturer's instructions (Vector Laboratories, Peterborough, UK). Sections were dehydrated and mounted before microscopic examination. These sections were then used to count ICAM-1-positive vessels in 25 consecutive microscopic fields at 400× magnification.

### Proliferation assays

CD4^+^ T cells were purified from splenocytes of *P. berghei* ANKA-infected mice by negative selection with Dynabeads following the manufacturer's instructions (Invitrogen Dynal, Norway). CD4^+^ cells suspended in complete RPMI-1640 medium, 5% FCS, were seeded in 96-well plates (5×10^5^ cells/ml). Naïve syngeneic splenocytes irradiated to 3000 rads were added as antigen presenting cells at a density of 2×10^6^ cells/ml. Cells were then stimulated in triplicate for 3 days with *P. berghei* ANKA lysate (20 µg/ml) or anti-CD3 (5 µg/ml). Cells cultured in medium alone were used as background controls. [Methyl-^3^H]-thymidine (2 µCi/well, 5 Ci/mmol, Amersham UK) was added 16 h before harvest and radioactivity was measured in a betaplate counter.

### *P. berghei* ANKA lysate preparation

Blood collected from *P. berghei* ANKA infected mice were diluted 1∶2 in RPMI-1640 medium and passed through a Whatman CF-11 cellulose column. The erythrocytes were eluted by washing the column with 2 volumes of medium. The purified erythrocytes were centrifuged at 400×g for 5 min and trypsinized for 10 min at 37°C to remove mouse antibodies bound to cell membranes. After washing 3 times with RPMI-1640 medium, the erythrocytes were lysed with PBS-0.05% saponin and centrifuged at 10000 rpm for 10 min. The pellet was washed and resuspended in PBS. The parasites were disrupted by 5 cycles of freezing-thawing and centrifuged for 5 min at 400×g. The supernatant was collected and its protein content was determined using a protein assay (BioRad, CA). Lysate aliquots were stored at −20°C until use.

### ELISA for IFN-γ detection

Ninety-six-well plates were coated with capture antibody (R4-6A2) by overnight incubation at 4°C in Phosphate Buffer pH 9.0. Plates were then blocked with 1% BSA for 1 h at 37°C. Culture supernatants or serum samples were tested in triplicates by overnight incubation at 4°C. Plates were then incubated for 2 h at 20°C with the biotinylated antibody (XMG1-2) and then for 1 h with streptavidin-peroxidase conjugate (Pierce, Rockford, IL). Bound complexes were detected with tetramethyl-benzidine (KBL, Gaithersburg, MD) and H_2_O_2_. Absorbance was read at 450 nm. Cytokine concentration was calculated using recombinant IFN-γ for the preparation of standard curves.

### Adoptive transfer of T cells specific for parasite-expressed antigens

*P. berghei* transgenic parasites expressing MHC I and MHC II-restricted epitopes from chicken OVA (OVA_257–264_, H-2K^b^-restricted; OVA_323–339_, I-A^b^ and IA^d^-restricted) linked to GFP (PbTG), or GFP alone (PbG) were used for analysis of antigen specific T cells [17]. Both lines also express a selection cassette encoding a mutated form of the dihydrofolate reductase synthase gene of *T. gondii* that confers resistance to pyrimethamine. Mice were challenged with 1×10^6^ PbTG or PbG pRBC and treated with pyrimethamine (10 mg/kg) in the drinking water to maintain transgene expression. OT-I CD8^+^ and OT-II CD4^+^ T cells were purified from TCR transgenic (Ly5.1^+^) mice by negative selection as described [17]. In some experiments, purified CD4^+^ and CD8^+^ T cells were resuspended at a density of 1×10^7^cells/ml in PBS containing 5 µM CFSE (Molecular Probes, Eugene, OR). Cells were incubated at 37°C for 10 min and washed 3 times with complete RPMI medium, 5% FSC. Ly5.2^+^ mice were injected with 2×10^6^ CD4^+^ and CD8^+^ T cells 2 days before challenge with transgenic parasites. Splenocyte suspensions were prepared on day 5 p.i. and T cells specific for parasite-expressed OVA were detected by staining with PE-anti-Ly5.1, PerCPCy5.5-anti-CD8 or APC-anti-CD4. CFSE staining was assessed by flow cytometry on gated CD4^+^ or CD8^+^ Ly5.1^+^ cells.

### Statistical analysis

Normal distributions of data sets were assessed using the Kolmogorov-Smirnov test. A Student's t-test was used for data evaluation of data sets with confirmed normal distribution. All other data sets were evaluated using a Mann-Whitney nonparametric test. Differences in mortality rates of *P. berghei* infected mice were assessed by Cox-Mantel logrank analysis.

## Supporting Information

Figure S1 Lack of IP-10 reduces systemic IFN-γ responses to malaria.
(1.06 MB TIF)

Video S1 Cerebral (pial) postcapillary venule of a naïve control C57BL/6 mouse. Rhodamine 6G was used to stain interacting cells.
(5.92 MB AVI)

Video S2 Cerebral (pial) postcapillary venule of a *P. berghei* ANKA-infected mouse treated with an isotype control antibody. Rhodamine 6G was used to stain interacting cells and video was captured on day 5 p.i.
(5.07 MB AVI)

Video S3 Cerebral (pial) postcapillary venule of a *P. berghei* ANKA-infected mouse treated with anti-IP-10 monoclonal antibody. Rhodamine 6G was used to stain interacting cells and video was captured on day 5 p.i.
(5.11 MB AVI)
