# Supplementary figures and images for: IP-10-Mediated T Cell Homing Promotes Cerebral Inflammation over Splenic Immunity to Malaria Infection
Source: PLoS Pathog. 2009 Apr 3;5(4):e1000369. doi: 10.1371/journal.ppat.1000369 (PMC2658824; doi:10.1371/journal.ppat.1000369)

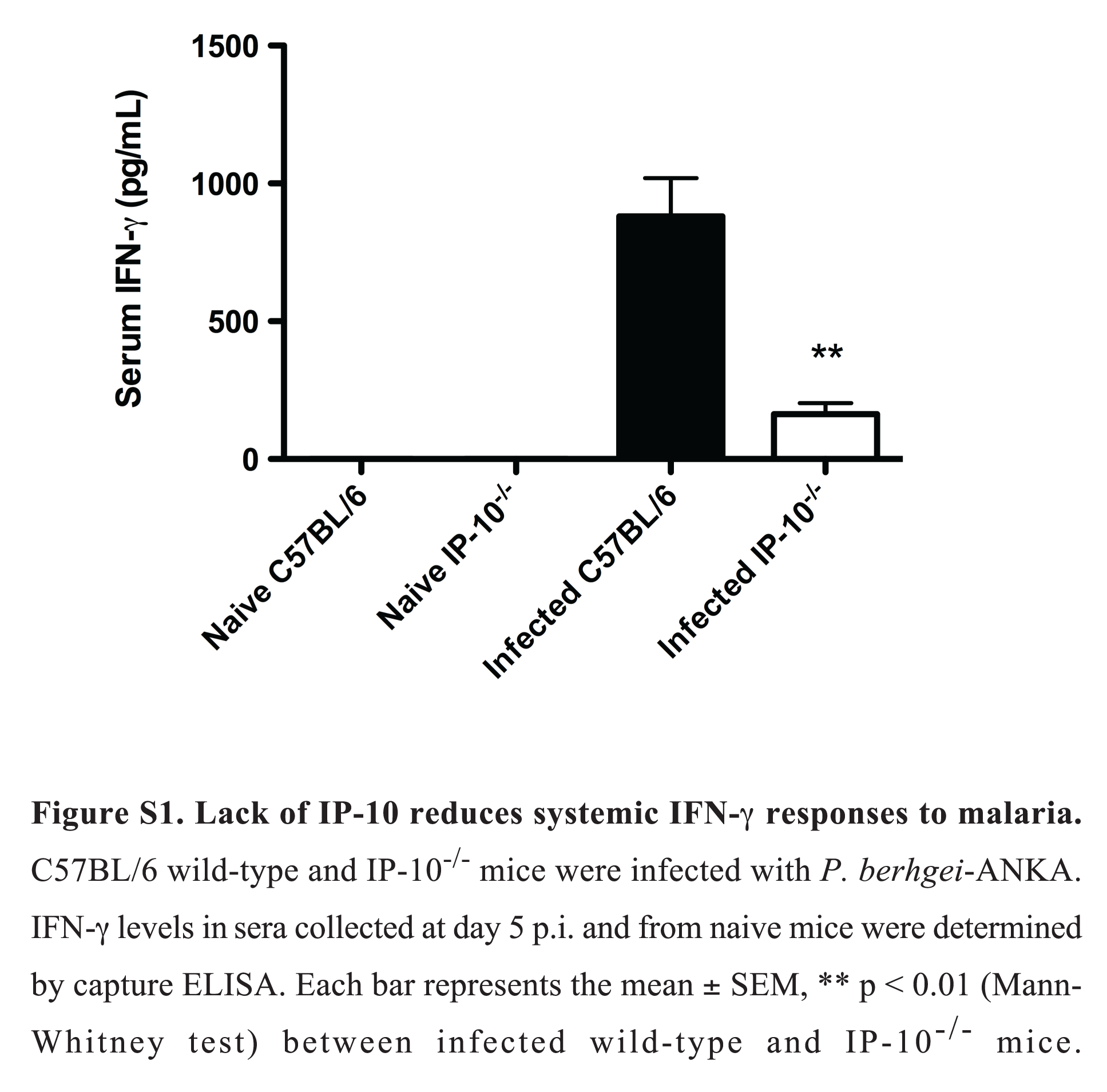

Supplement: Figure S1 — Lack of IP-10 reduces systemic IFN-γ responses to malaria. (1.06 MB TIF) [file ppat.1000369.s001.tif]
